# Access to scholarly publications in the global North and the global South—Copyright and the need for a paradigm shift under the right to science

**DOI:** 10.3389/fsoc.2023.1277292

**Published:** 2023-11-15

**Authors:** Klaus D. Beiter

**Affiliations:** Faculty of Law, North-West University, Potchefstroom, South Africa

**Keywords:** copyright, right to science, scholarly publications, adequacy for science, scholarly knowledge commons, global South, extraterritorial state obligations, commercial scholarly publishers

## Introduction

Article 15(1)(b) of the International Covenant on Economic, Social and Cultural Rights (1966) (ICESCR) protects the right of everyone “to enjoy the benefits of scientific progress and its applications” (the right to science). In the digital age, tensions between copyright and the unencumbered dissemination of, access to, and use or reuse of scholarly publications, impeding the conduct of science, but also citizens' access to scholarly publications as a benefit of scientific progress, have been growing continuously. These tensions have been felt acutely in the global South where digital locks and fences have particularly devastating effects. There is an increasing awareness of the need to resolve these tensions. Current reforms are thus being undertaken at perhaps these three levels: firstly, there is the endeavor to recognize the legitimacy of a “secondary publication right” (a circumscribed right of academic authors to self-archive their papers for open access); secondly, computational methods of research such as text and data mining (TDM) are sought to be considered a permissible use in relation to copyright-protected collections, and, thirdly, mechanisms are being developed to decrease the costs for researchers in developing countries to publish open access in the journals of, and access research published in, the developed world.

However, if science is a human right – that is, if there is a right of scientists to conduct research and a right of citizens to access (inter alia) scholarly publications as a benefit of science – then something else, “much more,” would be required. Then it does not suffice to merely scratch at the surface of current scientific copyright practices. If science is a human right, then science must also be allowed to function “adequately” – the concept of “science adequacy” to be examined further below – so that science can actually produce benefits, whether scientific knowledge, technology, general enlightenment, or democracy, etc., for citizens. Focusing on the copyright context, science as a human right – thus the argument made in this contribution – would require three much more drastic measures.

*Firstly*, the political economy of the current, increasingly commercialized, science system – of which economic copyright (or intellectual property in general) is a constituent element – would need to be fundamentally questioned. It would be necessary to move toward “another” science, that is, a science that is not primarily driven by instrumental and profit motives, as these damage science. *Secondly*, following from the previous point, science would need to be *genuinely* open. Hence, it would be necessary to construct a “*true”* scholarly knowledge commons, which transcends practiced forms of “green” and “gold” open access (concepts discussed below), obviates the need for complex legislation aimed at wrenching the right to perform TDM from the commercial publishers, and demotes the economic rights of copyright, but strengthens the moral ones, so that these can properly facilitate reputation building by scientists and encourage a broad public discourse on scientific findings. *Thirdly* – although strictly already part of the former point, but so important as to warrant separate mentioning – it would need to be ensured that the envisaged scholarly knowledge commons is “global” in scope. This is achieved by properly identifying, and respecting, the still largely ignored international dimension to the right to science. This dimension is based on group rights of development and international solidarity, and extraterritorial state obligations under the right to science. Science in the global South needs to be protected against global copyright law that, in its current design and interpretation, is essentially a product of the North, and against the international commercial scholarly publishers based and enclosing scientific knowledge in the global North.

The suggestions made in this contribution ultimately propose that the right to science should serve to *properly* realize access to scholarly publications for all, in the global North and the global South, by moving toward a world where commercialized science, economic copyright, and commercial scientific publishers play a reduced role. As it were, a paradigmatic shift is required under the right to science.

## Copyright as an impediment to open science

Article 2(1) of the Berne Convention for the Protection of Literary and Artistic Works (1971) requires copyright protection to be available for scientific works. Traditionally, the copyright in scientific works is held by academics, not the research institutions that employ them (Caso, 2020, p. 28; Bellia and Moscon, 2021, p. 5–11). Publishers customarily expect researchers to transfer to them the copyright in their scientific writings. Especially in a context where at least 60 percent of academic journals (thus figures from 2006) are commercially owned or published (Morrison, 2009, p. 37), copyright has become the cornerstone of a lucrative business model, with dire consequences for science. Copyright has come to impede the dissemination of, access to, and use or reuse of scientific knowledge. Hence, publishing contracts limit scientists' rights to republish, distribute or communicate, or produce derivative versions of their own works (Guibault, 2011, p. 148–151). Furthermore, access is restricted for both scientists in research institutions and ordinary citizens seeking access to existing research findings. While library budgets remain stagnant, publishers have been pursuing “an aggressive policy regarding prices” (Weingart, 2017, p. 96). Subscription fees for journals have outpaced inflation by over 250 percent in the last 30 years (Electronic Frontier Foundation). Libraries obviously can only offer such (limited) content as is affordable to them. Insofar as use or reuse is concerned, the Berne Convention in its current 1971 version does not expressly allow members to adopt limitations and exceptions to copyright protection to benefit research. It was considered that the Article 9(2) exception to the general reproduction right of Article 9(1) would sufficiently cater to all conceivable research needs. In the digital era, the way science is conducted has changed, however. Text and data mining (TDM), for instance, makes it possible to browse, analyse, and manipulate huge amounts of digital text or data within seconds. It is not clear whether current copyright limitations and exceptions would permit such use with respect to the accessible collections of a research institution (Flynn et al., 2020). One could potentially view TDM as an instrument of “massive infringement” of copyright (Reichman and Okediji, 2012, p. 1412, 1426–1428). Also the more recent World Intellectual Property Organization (WIPO) Copyright Treaty (1996) (WCT), which sought to adapt copyright to the digital age – and which, in its preamble, recognizes the need to protect the public interest in research – does not create specific limitations and exceptions for research.

All these problems have become more acute in the digital age. Hence, digital works are licensed, not sold to libraries, permitting the charging of fees and exercise of control on a perpetual basis. So-called technological protection measures (TPMs) further make it possible to absolutely control dissemination, access, and use or reuse. As has been said, digitization “creates the digital dilemma in copyright of scientific works: digitalisation allows maximum access and at the same time maximum control” (Peukert and Sonnenberg, 2017, p. 218). In the analog era, the doctrine of first sale still permitted unencumbered lending by libraries (Reese, 2003, p. 577–578). There also existed flexibility in the application of limitations and exceptions. In the digital context, all these freedoms are a thing of the past now.

For a number of reasons, the restrictions of copyright weigh particularly heavy in the sphere of science. Scholarly communication is crucial to the functionality of science. It is a part of the science verification mechanism in terms of which scientists are to test and validate the knowledge claims made by other scientists, it enables such scientists to rely on this knowledge in the creation of yet new knowledge, it facilitates reputation-building by scientists, thus motivating them to publish and make known their findings, and it yields access to research findings for society at large (Merton, 1973a, p. 273–275, 277–278; 1973b; Ziman, 2003, p. 33–36, 40–44). Scientific works are, moreover, not “substitutable” (Weingart, 2017, p. 100). This is so because scientific method and respect for the accepted moral rights of attribution and integrity of authors require access to the specific scientific source concerned, engagement with that recorded rendering of knowledge, and citation of exact page numbers.

In the light of the deficiencies of the subscription or “paywall” model of access to scholarly publications, there is a move toward open access (OA). Under UNESCO's 2021 Recommendation on Open Science, OA signifies that content is openly available online, and accessible and reusable for everyone free of charge and without undue restrictions, immediately or as quickly as possible (after publication) (UNESCO Recommendation, 2021, paras. 6–8). In practice, two forms of OA have crystallized for scholarly publications: gold OA publishing and green OA self-archiving [para. 7(a)]. In the golden path, the author grants a publisher the non-exclusive license to publish a work and communicate it to the public. The author retains the copyright and may grant an open content license facilitating wide use or reuse of the work by the public. The principle of “the author pays” (thus not the library) prevails in this path. This often requires copyright to be redeemed at exorbitant prices charged by the commercial publishers in the form of so-called “Article Processing Charges” (APCs) (on average almost a thousand US dollars) (Solomon and Björk, 2012). In the green path, directed at wholly or partially publicly funded subscription articles, and books for which the author does not wish to be paid, authors deposit their works in an OA subject or institutional archive. Unless legislation confers this “secondary publication right,” authors' right of deposit needs to be negotiated with publishers. Publishers often set embargo periods of 6 to 12 months. Furthermore, they do not allow the archiving of articles in their published format (often only a pre-print) – which means that, for citation purposes, the actual article will yet have to be consulted. In this path, access to archived articles is *gratis* (ie, access is cost-free for the user), but usually not *libre* (ie, use or reuse is not free from copyright/licensing restrictions).

Overall, the publishing model in science, rooted in copyright, is “grotesque” (Hilty, 2005, p. 123): the public pays more than two thirds of all research in higher education institutions (OECD, 2017, p. 100) (in fact, the public pays thrice: for the research, for the publication, and for library usage under collective agreements) (Guibault, 2011, p. 138), authors do not receive money for their articles (authors write “for” reputation, not money) (Merton, 1973b), and the five big publishers dominating the global science publication market (Larivière et al., 2015), despite the move to less costly desk-top publishing (Van Noorden, 2013), charge ever higher prices and achieve profits of up to 40 percent (Morrison, 2009, Ch. 3). A report produced by Deutsche Bank in 2005 finds the multiple-pay model “bizarre,” journals' working capital requirements to be “minimal,” and the professional publishers, overall, to “add little value to the research process” (Klein, 2019).

## Copyright as an obstacle in the global South

Research findings need to be validated *universally* before they can be considered to constitute new knowledge (Merton, 1973a, p. 270–273; Ziman, 2003, p. 36–38). In other words, the global South would have to be fully engaged in scholarly communication processes across borders toward this end, for its own findings to achieve universal validity and those of the global North, as well. This presupposes the free flow of scientific information on a transboundary scale. “[U]nduly strong copyright rules” would not only impede this endeavor, but – as already pointed out by the famous 2002 Report of the United Kingdom Commission on Intellectual Property Rights – they would also prevent affordable access to scientific works essential for development, in countries of the global South (CIPR, 2003, p. 18). As is well known, exclusion has exponentially aggravating effects in developmental contexts. The report thus refers to the problem of paywalls for development (p. 100). Similarly, it states that development through research is stifled by TPMs, supplemented by contract law allowing legitimate uses of works to be excluded, these measures effectively restricting forms of fair use (p. 100). A recent analysis highlights that outdated copyright laws in many countries, notably also in countries of the developing world, fail to adequately cater to legitimate research uses, including TDM (Fiil-Flynn et al., 2022). Furthermore, where differential pricing in licensing is applied to benefit developing countries, it has been observed that current practices ultimately produce “a system that operates at the edge of affordability for all players” (Karaganis, 2018, p. 12). Moreover, insofar as OA publishing is concerned, UNESCO's recent Recommendation on Open Science notes that “increased costs for scientists and high article processing charges associated with certain business models in scientific publishing … may be causes of inequality for the scientific communities around the world” (UNESCO Recommendation, 2021, para. 20). As it were, neither researchers nor their institutions in the global South can afford the APCs charged by many international journals.

## Copyright reforms: Remaining within the current paradigm

To be sure, the paradigm referred to here is that which insists on an intermediary between scientist and audience, responsible for producing products that capture the scientist's results and for then distributing them. It is based on granting economic copyright, or its de facto monopoly, as notably in the case of gold OA, to scholarly publishers, many of these nowadays powerful commercial corporations. Such arrangements – meaningful (if non-abusive) in the analog age, where physical books or journals needed to be produced, whose production required technical skill, machinery, and material resources – have meanwhile become embedded in an extensively “commercialized” science system (on such “industrialization” of science, see Ziman, 2003, p. 77–79).

With the rising awareness that digital copyright increasingly obstructs the unencumbered dissemination of, access to, and use or reuse of scholarly publications, various reforms of copyright have been suggested, and to various degrees also been implemented. As regards authors' rights, it has been suggested that author-publisher standard contracts (or contract law as such) should be redesigned to ensure that no assignment of copyright takes place and that authors' rights of dissemination and use or reuse are preserved (Guibault, 2011, p. 161–162). Concerning the prices libraries are charged for access to subscription publications, price control could be implemented. A tribunal could find fees to be unreasonable (Reichman and Franklin, 1998, p. 930) or anticompetitive (Hilty, 2009, p. 641 ff). Secrecy clauses in licensing agreements could be forbidden to ensure transparency and prevent price discrimination (Taubert, 2017, p. 89). To facilitate legitimate research uses of scholarly materials, there is a need for, ideally, a comprehensive research exemption, benefiting students, researchers, and libraries, permitting commercial and non-commercial use and reuse of materials for all recognized scientific purposes, such as reproduction, communication to the public, adaptation, storage, and so on, subject to a broad fairness test (Reichman and Okediji, 2012, p. 1439–1441). This should specifically cover computational uses, including TDM (Flynn et al., 2020). The European Union has adopted a Directive in 2019, obliging E.U. members to provide for an exception permitting TDM for the purposes of scientific research (Directive, 2019/790, Art. 3). It has been stated that copyright law should, as of right, permit digital lending/communication of e-books by libraries (Spedicato, 2016, p. 156–157, 162–165). The European Court of Justice has, in 2016, in the case of *Vereniging Openbare Bibliotheken v. Stichting Leenrecht*, held that also digital books are subject to the E.U.'s (hitherto “analog”) book lending regime, permitting libraries to lend these to users, provided authors are remunerated (ECJ, 2016). Copyright law should further allow remote access to library collections (Statement on Copyright and Proposal of a Waiver, 2021). The contractual restriction or exclusion of limitations and exceptions for research should be forbidden (Reichman and Franklin, 1998, p. 929–931, 946; Hugenholtz, 2000, p. 81; Reichman and Okediji, 2012, p. 1447). Under the E.U.'s 2019 Directive, contractual provisions contrary to the TDM exception for research are thus unenforceable [Directive, 2019/790, Art. 7(1)]. Because TPMs easily obstruct proper research, they should, as far as possible, be avoided as regards scientific literature (Hugenholtz, 2000, p. 81). As for green OA, it has been suggested that legislation should grant authors an inalienable right to self-archive (Scheufen, 2015, p. 155; Bellia and Moscon, 2021, p. 13–14, 17). A 2022 study for the E.U. accordingly recommends that the E.U. legislator should introduce an E.U.-wide secondary publication right (Angelopoulos, 2022, p. 55). Countries such as the Netherlands and Germany have already, by way of legislation, introduced such a right for cases where a paper has at least in part been publicly funded (Visser, 2015). As for gold OA, it has been stated that APCs should be fair and borne by research institutions or other funders (Beiter, 2022, p. 169, 191). Hence, if copyright reform efforts, at the moment, focus most significantly on, firstly, statutory TDM exceptions for research and, secondly, statutory secondary publication rights, then a third sphere of change is that which seeks to develop mechanisms to decrease the costs for researchers in developing countries to publish open access in the journals of, and access research published in, the developed world. Publishing companies thus apply differential pricing for their products favoring developing states (Helfer and Austin, 2011, p. 336), or offer full or partial waiver schemes for APCs benefiting scientists in poorer countries (Tennant et al., 2016, p. 13). However, these are all *voluntary* measures adopted by publishers.

Under the three-step test of international copyright law, first laid down in Article 9(2) of Berne, repeated in Article 10 of the WCT, and now made internationally enforceable in the World Trade Organization under Article 13 of the Agreement on Trade-Related Aspects of Intellectual Property Rights (1994) (TRIPS), all the above research limitations and exceptions would have to be assessed for whether they could be said to be confined to, firstly, certain special cases, which, secondly, do not conflict with a normal exploitation of the work, and, thirdly, do not unreasonably prejudice the legitimate interests of the right-holder. Without examining this here in any detail, most of the above limitations and exceptions could be, and have been, argued to meet the demands of the three-step test. For instance, the above study for the E.U. holds that the secondary publication right, if limited to articles that are publicly funded, would likely comply with the three-step test (Angelopoulos, 2022, p. 55). In applying the three-step test, public interest considerations, and clearly also the fact that science has been proclaimed a human right, would need to be taken into account, especially as part of the third leg of the test (Geiger et al., 2008, p. 712). Article 7 of TRIPS ultimately considers the dissemination of knowledge an objective of TRIPS. Under Article 8, upstream science must be held to be linked to most or all socio-economic objectives that a state may promote in its IP policies. Moreover, research, as the WCT posits in its preamble, is in the public interest. All these provisions create a bridge between TRIPS and international human rights law (Yu, 2009, p. 1037, 1039).

Yet, while some of the above reforms would, or could, also be relevant as part of a system “beyond the current paradigm,” as proposed under the next headings, on their own, as part of this paradigm, they merely scratch at the surface of current, destructive, scientific copyright practices. Hence, the E.U.'s TDM exception does not apply to commercial research, which is in the public interest, too (Reichman and Okediji, 2012, p. 1440–1441; Flynn et al., 2020, p. 397). It does not facilitate access to copyrighted materials not part of the collection otherwise available in a specific library (Hilty and Richter, 2017, paras. 25–28). As for green and gold OA, the current arrangements lead to a system where scientific knowledge is dispersed over manifold archives. Some documents are available in their published format, others in a pre- or post-print format. Proper science, and the moral rights of copyright, require access to the formal version of record of a paper. Different conditions of use or reuse pertain to each document, and these are often unclear. Some information is available only after an embargo period. Library subscriptions and APCs remain expensive. Access for the global South, moreover, continues to be a matter of courtesy or discretion.

Is it not conceivable that, under a right to science, scientists and the general public may legitimately expect more? This is evident also from the fact that many of the above (arguments for) reforms have not primarily been framed from human rights. Some do rely on freedom of expression (Reichman and Franklin, 1998), scientific freedom (Hugenholtz, 2000), or the right to science (Beiter, 2022), to support their position; most, however, argue from the functionality or the needs of, or the public interest in, science, but not specifically from human rights. Human rights must, therefore, add something more substantial to the debate. Under the current paradigm, even as reformed, dissemination, access, and use or reuse remain significantly obstructed, while, simultaneously, enormous sums of public revenue, that could enhance science, flow to private intermediaries that add ever diminishing value to research (Reichman and Okediji, 2012, p. 1461). “Less innovation, not more, is the predictable result over time” (p. 1426). The right to science calls for a shift of paradigm.

## A paradigmatic shift: The right to science as the basis for “another” science

The right to science in Article 15(1)(b) of the ICESCR covers “the dissemination of scientific knowledge … within the scientific community and in society at large, including through publishing research findings.” This clearly follows from Article 15(2), which requires states parties to secure “the diffusion of science” (Shaheed, 2012, para. 48; Porsdam, 2022, p. 63–66). Moreover, under Article 15(3), states parties are to respect “freedom indispensable for scientific research.” As it were, the right to science may be held to cover a “right to research.” This has a conceptually more negative and a conceptually more positive side. The former is reflected in the right or freedom of scientists (but also others) “to do research,” the latter in the right of citizens “to enjoy access to the benefits of research” (Beiter, 2022, p. 162–177). The benefits of research include not only technology, but also the availability of useful scientific information, the capacity of citizens to make free and rational choices in life, a strengthened knowledge base to sustain democracy; and scholarly publications are a part of these benefits (CESCR, 2020, General Comment No. 25, paras. 6–8). Both dimensions of the right to research require the absence of undue restraints on the dissemination of, access to, and use or reuse of scientific publications which benefit scientists and ordinary citizens seeking access to research findings (Shaheed, 2012, para. 74(o); UNESCO Recommendation, 2017, Preamble, Recital 4(c); CESCR, 2020, General Comment No. 25, para. 49). OA would constitute the ideal solution in this regard (Skre and Eide, 2013, p. 430–440; UNESCO Recommendation, 2021, paras. 6–8). Copyright limitations in science can, therefore, not be justified by mere reference to the fact that they sustain a scholarly publication industry as a sector of the economy, if it cannot be shown that that industry adds significant value to the scientific enterprise. The scholarly publication industry does not enjoy a “constitutionally enshrined” institutional guarantee (Peukert and Sonnenberg, 2017, p. 226). As juristic (not natural) persons, publishing companies can also hardly raise human rights claims to the protection of their IP rights under Article 15(1)(c) of the ICESCR (CESCR, 2006, General Comment No. 17, para. 7), which protects everyone's right to benefit from “the protection of the moral and material interests” resulting from the scientific (or other) works one has authored, or under the right to property, as protected in (especially regional) international human rights law, as companies' IP claims are not, or only weakly, rooted in human dignity, the fundamental value underlying human rights (Beiter, 2022, p. 136–140).

The “openness” norm is of such pivotal importance to the functionality of science that the category of intellectual property in science is, in principle, suspect. Nobody has explained this better than Merton in his seminal work on the normative structure of science:

The substantive findings of science are a product of social collaboration and are assigned to the community. They constitute a common heritage in which the equity of the individual producer is severely limited. … Property rights in science are whittled down to a bare minimum by the rationale of the scientific ethic. The scientist's claim to “his” intellectual “property” is limited to that of recognition and esteem… The institutional conception of science as part of the public domain is linked with the imperative for communication of findings. Secrecy is the antithesis of this norm; full and open communication its enactment. The pressure for diffusion of results is reenforced by the institutional goal of advancing the boundaries of knowledge and by the incentive of recognition which is, of course, contingent upon publication (Merton, 1973a, p. 273–274).

The reality, of course, is the enormous expansion of IP rights as part of an increasingly commercialized science system. Universities are now called upon to become commercial actors, to be able to finance their various activities. In the sphere of innovation, this has led to universities patenting publicly funded research, the extension of exclusive rights to even basic research results, and restrictions on access to knowledge for the research community, follow-on innovators, and the general public (Nelson, 2004, p. 455, 462; Plomer, 2015, p. 10–13). As for copyright: The ability of universities to attract finance, for example fee-paying students, depends on positions in university rankings. These significantly rely on citations of works produced by an institution's academic staff (e.g., 30% in the Times Higher Education World University Rankings 2023). In the sphere of academic writing, this factor nourishes the publish or perish ideology so destructive to science (Moosa, 2018). Copyright in the hands of the publishing industry has come to serve as the mechanism through which this artificial writing explosion can be profitably exploited (Beiter, 2022). The recent General Comment No. 25 on the right to science, drafted by the Committee on Economic, Social and Cultural Rights, the ICESCR's monitoring body, observes that “the excessive price of some scientific publications is an obstacle for low-income researchers,” and (implicitly) that copyright may pose “significant obstacles” for citizens wishing to access scholarly publications as a benefit of scientific progress (CESCR, 2020, General Comment No. 25, para. 61). The Committee's response is to call for “a balance” between IP rights and open access and the sharing of scientific knowledge (para. 62). The notion of balance, however, seems to rather opt for remaining within the current paradigm and to accommodate commercial copyright interests.

Robert Merton's writings and the drafting history of the founding instruments of international human rights law teach us that “disinterestedness,” and extensive scientific freedom, are the hallmarks of academic science (Merton, 1973a, p. 275–277; Merton and Barber, 2004; Schabas, 2015; Smith, 2020; Kinzelbach, forthcoming). They are the guarantors of path-breaking discovery and of scientists finding answers to the fundamental questions of mankind. As Dasgupta and David explain, to maintain the economic structure of science, to secure downstream innovation and technology development, a special role for upstream science in universities needs to be carved out, a space where science can flourish freely, unfettered by external norms (Dasgupta and David, 1994). In 1939, Flexner thus highlighted “the usefulness of useless knowledge” for scientific progress (Flexner, 1939). What we witness today, however, is the “industrialization” of academic science in the pursuit of so-called “impact” (Ziman, 2003, p. 77–79; Nelson, 2004; Moriarty, 2011), its “bureaucratisation” (new public management) in the endeavor of enhancing “productivity” (Power, 1999, p. 94–104; Ziman, 2003, p. 79–82; Lee and Walsh, 2022), and neoliberal economic goals being imposed on universities (Slaughter and Leslie, 1997; Nowotny et al., 2005; Rider et al., 2013).

What does this have to do with copyright? Instrumental conceptions in science may readily be implemented in a way that is concomitant with overregulation of science, hyperincentives, productivist and market-oriented agendas, and performatist behavior, all detrimental to science. It is these facets that create a (lucrative) market for scholarly publications in the first place, in which copyright serves to buttress the economic power of, and leads to a diversion of scarce public resources for research to, the scholarly publishing industry (Beiter, 2022). Currently, governmental and institutional research evaluation systems thus emphasize publication in journals with a high impact factor (IF) and researcher effort to be invested in enhancing publication output (Moosa, 2018, p. 1, 13, 76, 181; Bellia and Moscon, 2021, p. 11–13). High journal IFs, often the result of an abuse of peer review processes (Cope and Kalantzis, 2009, p. 44), can hardly predict scientific quality (Brembs et al., 2013), but essentially serve to boost journal prices (Weingart, 2017, p. 98). For researchers in the developing world – because most journals here are not indexed – IFs entail that, to be published in a journal with a (high) IF, researchers will have to write about problems of the developed world, in the languages of the developed world, and satisfy a readership in the developed world (Mahroum, 2016). Quantitative goals are a pillar of the destructive publish or perish ideology in science. “The only people who benefit from the intense pressure to publish are those in the publishing industry” (Colquhoun, 2011). The evidence now proves that this pressure impedes the discovery process (Park et al., 2023), spurs scholarly dishonesty, and results in the publication of papers of deteriorating quality (Moosa, 2018).

General Comment No. 25's commitment to disinterested science appears rather weak. It now formulates goals for science, still purposively omitted in the founding instruments of international human rights law. While “peace” and “human rights” are noble goals (CESCR, 2020, General Comment No. 25, para. 6), the formulation of goals for science reflects conceptions of “instrumental” or “useful” (Beiter, 2019), “ideologizable” (Smith, 2020), or “illiberal” (Kinzelbach, forthcoming) science. Rather: A necessary step to achieving the paradigm shift in scientific copyright is to reaffirm *academic* science. This is part and parcel of understanding the right to science as providing the basis for “another” science. This reaffirmation might be achieved by a reliance on the concept of “science adequacy,” modeled on the German law concept of “*Wissenschaftsadäquanz*,” but developed further (Beiter, 2019). This requires that all structures, arrangements, and decisions in the field of science would have to be such as would be “in the best interest of science and scholarship,” rather than serve political, economic, or social usefulness, or managerial efficiency. There needs to be respect for the intrinsic requirements of science, that is, autonomy, intuition, anarchy, inefficiencies, delay, and risk. A central role should be accorded to (active) scientists themselves (and not “managers” of whatever type), in organizing science, as scientists, by reason of their training and experience, understand the needs of science best (p. 286).

## “Another” science and a “true” scholarly knowledge commons

Very concretely, the paradigm shift requires science to be *genuinely* open. It would be necessary to construct a “*true”* scholarly knowledge commons. What would be the features of a true scholarly knowledge commons? Such a knowledge commons must entail free publishing for researchers (Beiter, 2022, p. 199). Overall, to safeguard public research resources, and to protect funders and institutions, there needs to be a reorientation toward affordable scholarly publishing (p. 199). The commons must provide extensive scope for the ability to perform TDM and reuse material. Research published in the commons should be the result of “slow science,” produced in a context where science evaluation, while focusing on quality, not quantity, plays a reduced role. The commons should refrain from relying on, and displaying, journal IFs or article metrics/altmetrics. Research should increasingly be presented in mega journals and huge subject archives, thus facilitating “coherence” in science. Mega journals have a very broad subject scope, judge articles only on scientific soundness (not impact), provide for a large editorial board of academic (not professional) editors, and are open access (Binfield, 2014, p. 158). Over time, there should be a few huge subject archives, encompassing all fields, owned by institutionalized science (Wehrmeijer, 2014, p. 71). Platforms could use open source software that offers authors a guided path from submission to publication. Datasets should accompany papers. Publications should feature lay summaries. Automated translation services should be available. Scientists will have to also address lay audiences and diversify their writing. Accepted “scientific” genres need to be expanded to include social media texts, blogs, articles in the lay press, or preliminary or negative results (Bartling and Friesike, 2014, p. 8–9). UNESCO's Recommendation on Open Science, whilst appropriately idealizing OA to scholarly publications, does not really offer a blueprint for realizing “genuine” OA. It endorses current gold and green OA, reflects an overall deference to IP rights protection, and largely accepts the status quo of a world of science in which commercial publishers will continue to play a dominant role (Beiter, 2022, p. 160–162).

How could a “true” scholarly knowledge commons be realized from the perspective of copyright? Perhaps there are three ways of accomplishing this, presented here from the less to the more far-reaching. *Firstly*, there could be a system of automatic OA of academics' scientific works through statutory (compulsory) licensing against fair compensation for publishers by research institutions and funders, reimbursement organized collectively (Willinsky, 2023). In this model proposed by Willinsky, in which copyright remains with authors, the public enjoys open access and rights of (free) use or reuse, including TDM (notably Ch. 6). Willinksy maintains that his model complies with the three-step test, as it applies to the limited cases of scientific works, envisages fair remuneration (determined by royalty judges), and advances science (pp. 145–146). This model retains the category of copyright and the traditional role of the commercial scholarly publishers, but limits economic abuse, whilst ensuring OA (p. 146). Willinsky specifically argues his case from the “right to science” in the U.S. Constitution, that is, Congress's power “To promote the Progress of Science and useful Arts” (ArtI.S8.C8.1) (p. 3). However, this collective model would clearly pose practical challenges if to be realized on an international level.

*Secondly*, there could be a move toward peer production by the scientific community itself (Reichman and Okediji, 2012, Part III; Skre and Eide, 2013, p. 446). The latter thus regains ownership over its articles, journals, books, and book series. The scientific community assumes managerial, editorial, quality control, production, and circulation functions in respect of publication. The existing publishers could, if and to the extent needed, provide remunerated technical services that support the endeavor, but they would not hold copyright (Reichman and Okediji, 2012, p. 1466). This vests in authors, who, under open content licenses, grant open access and wide rights of (free) use or reuse, including TDM, to the public. To make this model work, additional infrastructural, human, and technical resources would be needed by the research community. State funding could be available though, as the commercial publishers would no longer capture large sums of public revenue through copyright, and as, under “another” science, resources presently consumed by neoliberal science bureaucracy, would be released. This model need not be justified in terms of the three-step test because it constitutes a form of self-help by science (to be supported by the state). Ultimately, nobody can be forced to contract with a publisher.

*Thirdly*, as under the previous point, the scientific community could retain ownership of its research, published by way of peer production, but economic copyright in the sphere of science could be abolished altogether (Shavell, 2010; Moscon, 2015, p. 128–129; Beiter, 2022, p. 206–212). In the field of science, copyright does not fulfill the incentive function customarily ascribed to it. Academics are not paid for articles. Their motivation for writing lies in scientific reputation-building (Skre and Eide, 2013, p. 439; Eger and Scheufen, 2018, p. 10–11; Bellia and Moscon, 2021, p. 4). Regarding books, for which academics are often paid, the incentive function does not work either. The publication of academic books has been steadily declining (Savage and Olejniczak, 2022). Furthermore, the reward function of copyright can be satisfactorily substituted with adequate salaries, job security, and substantial academic freedom (Dasgupta and David, 1994, p. 513–515, 518; Skre and Eide, 2013, p. 439; Moscon, 2015, p. 101). It is important to emphasize, however, that authors' moral rights of attribution and integrity remain crucial to the functioning of science, and need to be strengthened. Moral rights are human rights (CESCR, 2006, General Comment No. 17, para. 13). Caso underlines that the right of attribution must again become “the engine of a public dialogue and of a dynamic relationship between individual contribution and collective advancement of knowledge” (Caso, 2020, p. 31). Nevertheless, abolishing economic copyright in the sphere of science cannot be realized under existing international copyright law, since, as pointed out, the Berne Convention requires copyright to be available for scientific works. This is, however, exactly where science as a human right, as will be explained below, could play a crucial role.

## The international dimension of the right to science: A “global” scholarly knowledge commons

By its nature, science is “one of the most international of all activities” (Chapman, 2009, p. 27). Science is a global public good (UNESCO Recommendation, 2021, paras. 13(b), 18). The dissemination of, access to, and use or reuse of scholarly information should be possible and facilitated across national borders. “Global science inclusiveness” signifies that no country or region, also not the global South, may be excluded from participation in what is ultimately a universal scientific enterprise [paras. 6, 13(b)]. Of crucial significance to the argument made in this contribution, a scholarly knowledge commons will only be a “true” scholarly knowledge commons, if it operates globally. It would have to be fully functional across borders and facilitate the free flow of knowledge for all, within the global North, within the global South, and between global North and global South.

By virtue of Article 15(4) of the ICESCR, calling on states parties to encourage and develop international contacts and co-operation in the scientific field, the international dimension of the right to science is already built into the very structure of Article 15. Based on group rights of development and international solidarity, with a legally binding basis in (inter alia) the ICESCR, and on extraterritorial state obligations (ETOs) to respect, protect, and fulfill human rights, collective and individual claims under the right to science, envisaging the enjoyment of unimpeded dissemination of, access to, and use or reuse of scientific knowledge *across borders*, can be identified (Beiter, 2022, p. 177–185). For lack of space, just ETOs will be considered here. According to the expert Maastricht Principles on Extraterritorial Obligations of States in the Area of Economic, Social and Cultural Rights of 2011, ETOs to respect, protect, and fulfill arise under all human rights, including the right to science (Maastricht Principles, 2011, Principle 3). ETOs create obligations for states, in appropriate circumstances, to safeguard and advance the human rights of those beyond their own borders.

ETOs to protect, for example, would require states to regulate and monitor the copyright-related conduct of “their” (home) publishing companies doing business abroad, where this affects the right to science abroad (De Schutter et al., 2012, p. 1141). Publishing companies could thus be required to grant the right to self-archive to researchers in all countries. They might be expected to apply differential pricing (Helfer and Austin, 2011, p. 336), or offer waiver schemes for APCs (Tennant et al., 2016, p. 13), benefiting institutions and researchers in developing states. “Subscription” agreements with libraries might have to cover not only access charges, but also fixed compensation for “all needed research uses” (Reichman and Okediji, 2012, p. 1467), and even APCs for researchers at the institution concerned, all built into the “subscription” price, to facilitate more transparent and affordable deals. Hence, many of the global South reforms undertaken within the existing paradigm, that at present are in the form of voluntary benefits granted, could be required to be given compulsory status.

ETOs to respect, protect, and fulfill, when read in conjunction with domestic state obligations under the right to science, imply rights of scientists and citizens to seek, receive, use or reuse, impart, or publish scholarly knowledge across borders. As it were, one state must allow, facilitate, and contribute to knowledge exportation (under ETOs), the other must allow, create capacity for, and actively support knowledge importation (under domestic state obligations) (Chapman, 2009, p. 28). Of particular significance in the present context are ETOs to facilitate. The Maastricht Principles thus require states to work together in the endeavor of creating an international enabling environment conducive to the universal fulfillment of economic, social, and cultural rights (Maastricht Principles, 2011, Principle 29). This would cover facilitating conducive conditions for the international transfer of scientific knowledge under the right to science. Consequently, states are obliged to negotiate IP treaties or adopt domestic IP regimes in a way, or direct their efforts in the World Intellectual Property Organization or the World Trade Organization toward, fostering the right to science, and international scientific knowledge transfer, globally (CESCR, 2020, General Comment No. 25, para. 83). In developmental contexts, copyright limitations and exceptions should be allowed to permit much wider research uses or reuses [CIPR, 2003, p. 104; Proposal for Treaty of Access to Knowledge, 2005, Art. 3-12(a)(3)]. Ultimately, states would be required to facilitate the implementation of a suitable model (see the previous heading) aimed at realizing a scholarly knowledge commons that operates globally. In this sense – and accepting the priority of human rights and ETOs over other international law (see this author's extensive argument, Beiter, 2020, Part VIII) – the right to science might even oblige states to amend the Berne Convention to abolish economic copyright in the sphere of science – to help accomplish the much-needed paradigm shift. The CESCR's General Comment No. 25 is not courageous enough to say this.

## Author contributions

KB: Writing—original draft.
